# Typical epidemiology of respiratory virus infections in a Brazilian slum

**DOI:** 10.1002/jmv.25636

**Published:** 2019-12-09

**Authors:** Luiz Gustavo Bentim Góes, Rodrigo Melim Zerbinati, Adriana Fumie Tateno, Andrea Vieira de Souza, Fabian Ebach, Victor M. Corman, Carlos Alberto Moreira‐Filho, Edison Luiz Durigon, Luiz Vicente Ribeiro Ferreira da Silva Filho, Jan Felix Drexler

**Affiliations:** ^1^ Institute of Virology, Charité‐Universitätsmedizin Berlin, corporate Member of Freie Universität Berlin Humboldt‐Universität zu Berlin, and Berlin Institute of Health Berlin Germany; ^2^ Laboratório de Virologia Clínica e Molecular, Departamento de Microbiologia – ICB‐II Universidade de São Paulo São Paulo Brazil; ^3^ Institute of Virology University of Bonn Medical Center Bonn Germany; ^4^ Instituto de Ensino e Pesquisa Hospital Israelita Albert Einstein São Paulo Brazil; ^5^ German Center for Infection Research (DZIF), associated partner site Charité Berlin Germany; ^6^ Departmento de Pediatria Faculdade de Medicina da Universidade de São Paulo São Paulo Brazil; ^7^ Instituto da Criança Hospital das Clínicas da FMUSP São Paulo Brazil; ^8^ Martsinovsky Institute of Medical Parasitology, Tropical and Vector‐Borne Diseases Sechenov University Moscow Russia

**Keywords:** acute respiratory infection, Brazil, epidemiology, real‐time polymerase chain reaction, respiratory tract infections, slum, viruses

## Abstract

Host population size, density, immune status, age structure, and contact rates are critical elements of virus epidemiology. Slum populations stand out from other settings and may present differences in the epidemiology of acute viral infections. We collected nasopharyngeal specimens from 282 children aged ≤5 years with acute respiratory tract infection (ARI) during 2005 to 2006 in one of the largest Brazilian slums. We conducted real‐time reverse transcription‐polymerase chain reaction (RT‐PCR) for 16 respiratory viruses, nested RT‐PCR‐based typing of rhinoviruses (HRVs), and collected clinical symptoms. Viruses were common causes of respiratory disease; with ≥1 virus being detected in 65.2% of patients. We detected 15 different viruses during 1 year with a predominance of HRV (33.0%) and human respiratory syncytial virus (hRSV, 12.1%) infections, and a high rate of viral coinfections (28.3%). We observed seasonality of hRSV, HRV and human coronavirus infections, more severe symptoms in hRSV and influenza virus (FLU) infections and prolonged circulation of seven HRV clusters likely representing distinct serotypes according to genomic sequence distances. Potentially unusual findings included the absence of human metapneumovirus detections and lack of typical FLU seasonal patterns, which may be linked to the population size and density of the slum. Nonetheless, most epidemiological patterns were similar to other studies globally, suggesting surprising similarities of virus‐associated ARI across highly diverse settings and a complex impact of population characteristics on respiratory virus epidemiology.

## HIGHLIGHTS

- Large epidemiological study of respiratory viruses in children from one of the biggest Brazilian slums using sensitive molecular PCR assays
- Viruses are common causes of respiratory disease in pediatric patients from a Brazilian slum
- 15 distinct viruses circulate in the Brazilian slum, with highest prevalence of rhinoviruses and human respiratory syncytial virus
- The epidemiology of virus‐associated respiratory infections presents surprising similarities worldwide despite drastic differences in socioeconomic status and population characteristics

## 1 INTRODUCTION

Acute respiratory tract infections (ARI) are the main cause of morbidity and mortality among children aged <5 years in the developing world.^1^ Respiratory viruses cause up to 80% of ARI.^2^

Respiratory viruses are spread via three different transmission routes: contact, droplet, and aerosol transmission.^3^ Host population size, density, immune status, age structure, and contact rates affect the transmission patterns of viruses causing acute predominantly self‐limiting infections, such as respiratory viruses.^4^ Residents of resource‐limited communities such as slums may be particularly vulnerable to virus‐associated ARI. Hypothetically, virus transmission may be facilitated in these dense populations, characterized by frequent interindividual contact, crowded housing, improper sanitation systems, poor education, and poor nutritional status, exemplified by inversely correlated influenza virus prevalence and family income in a study from Bangladesh.^5^

The United Nations define slums as human settlement areas that combine the following attributes: lack of basic services as sanitation and water sources, substandard housing or illegal and inadequate building structures, overcrowding and high density, unhealthy living conditions and hazardous locations, insecure tenure; characterized by irregular or informal settlements, poverty, and social exclusion.^6^

Close to 880 million people worldwide reside in urban slums, and this number is expected to double by 2025.^7^ Nonetheless, little is known about how and whether disease patterns in urban slums differ from affluent settings.^8^ Pivotal epidemiological studies conducted in slum cohorts from Bangladesh and Kenya highlighted the importance of respiratory viruses in these communities.^5^, ^9^, ^10^

In Brazil, 11.4 million people, nearly 6% of the country's population live in slums (https://ww2.ibge.gov.br/home/). Data on virus‐associated ARI from slum communities, particularly from Brazil, are scarce. In a study based on clinical symptoms, ARI in children inhabiting a Brazilian slum were very frequent, representing 50% of child infections events through a 1‐year period.^11^ A study combining clinical and virological data reported ARI symptoms in 60% of children inhabiting another Brazilian slum and in 35% of these children, the virus was isolated by cell culture.^12^ Moreover, surprisingly high virus or bacterial detection rates (up to 85%) were observed in a study conducted in northeastern Brazil in children under 5 years from low‐income families presenting with ARI.^13^

We previously found that virus‐associated ARI showed similar epidemiological patterns between a rural African and an urban European setting, including an overall similar spectrum of viruses, age associations and seasonal fluctuation despite drastic differences of socioeconomic status (SES) and climatic patterns.^14^ Hypothetically, lower SES in the African setting may have been equaled by higher population density in the European setting, both of which likely facilitate virus transmission.

**Figure 1:**
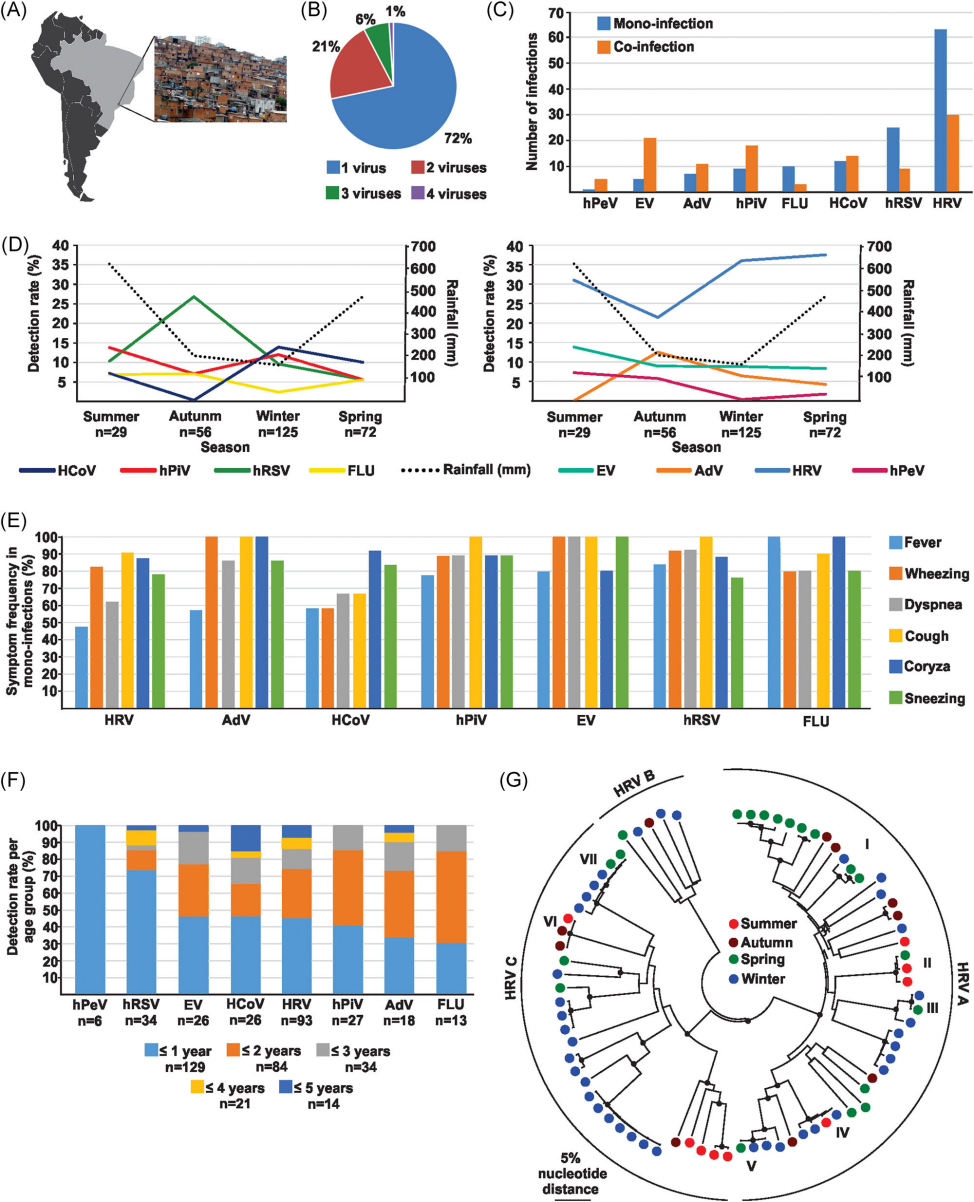
Epidemiology of respiratory viruses in a Brazilian slum. A, Paraisópolis, adapted under a creative commons license from: https://commons.m.wikimedia.org/wiki/File:Paraisopolis_sao_paulo.jpg#mw‐jumpto‐license and freely available data from www.naturalearth.com. B, Percentage of monoinfections and coinfections. C, Percentage of monoinfections and coinfections by the virus. D, Seasonality of enveloped (left) and non‐enveloped viruses (right) shown separately for clarity of presentation. E, Symptom frequency in monoinfections. Only one monoinfection was observed for human parechovirus and clinical data are not shown for clarity of presentation. F, Virus detection by age group. G, Rhinovirus phylogeny representing the study period. Species are given next to circles. Roman letters I‐VII designate HRV clusters occurring over more than one season. Black dots at internal nodes represent support of grouping higher than 75% from 1000 bootstrap replicates. AdV, adenovirus; EV, enterovirus; FLU, influenza A and B viruses; HCoV, human coronaviruses 229E, NL63, OC43 and HKU1; hPeV, human parechovirus; hPIV, human parainfluenzaviruses 1 to 4; hRSV, human respiratory syncytial virus; HRV, human rhinoviruses A‐C

Here, we investigated 16 respiratory viruses by real‐time (reverse transcription)‐polymerase chain reaction (RT‐PCR)–based methods in one of the biggest slums of Latin America, namely Paraisópolis. This community is located within the urban area of São Paulo city, inhabited by 89.000 residents in a 1.5 km^2^ area (approximately 59.300 inhabitants/km^2^) and distributed in 21 thousand dwellings including shacks, masonry buildings, townhouses and older and solid buildings^15^ (Figure 1A).

## 2 MATERIALS AND METHODS

Nasopharyngeal aspirates were collected longitudinally from February 2005 to February 2006 from 282 infants and children. Inclusion criteria were patient age equal or below 5 years and presence of at least 2 symptoms of ARI including cough, coryza, sneezing, dyspnea, wheezing and/or fever. From 282 patients, 12 presented with 2 symptoms (4%), 37 presented with 3 symptoms (13%), 52 presented with 4 symptoms (18%), 84 presented with 5 symptoms (30%) and 95 patients presented with all 6 symptoms that were recorded (34%); for 2 patients we lacked clinical data. From the 282 included patients, 22 presented underlying disease (8%). More specifically, a total of 21 patients had heterogeneous symptoms of heart disease, 16 of which tested positive for at least one virus and 5 of which tested negative. One patient that tested negative for all respiratory viruses was infected with HIV, none presented cystic fibrosis or tuberculosis. The underlying disease did not lead to exclusion from this study. Samples were collected from the pediatric hospital Darcy Vargas (n = 157) and the Paraisópolis Basic Health Unit (n = 125), in the Paraisópolis area, São Paulo, Brazil. The gender‐balanced cohort comprised 136 males and 146 females at a median age of 14 months (interquartile range, 7‐24). Samples per season were: Summer, n = 29 (December 21st‐March 19th, frequent rains, relatively higher temperature); autumn, n = 56 (March 20th‐June 20th, dry, decreasing temperature); winter, n = 125 (June 21st‐September 21st, dry, cold); spring, n = 72 (September 22nd‐December 20th, humid, increasing temperatures). Ethical approval was obtained from the Hospital Israelita Albert Einstein research ethics committee (protocols 10/1291 and 191/3). Informed consent was obtained from parents or guardians of children. Samples, symptoms and demographic data were collected by medical personnel upon medical consultation.

Laboratory analyses were done using real‐time RT‐PCR assays for 16 respiratory viruses including enteroviruses (EVs), human rhinoviruses (HRVs), human parainfluenzaviruses 1 to 4 (hPiV1‐4), human coronaviruses (HCoV) 229E, NL63, OC43 and HKU1, human metapneumovirus (hMPV), human respiratory syncytial virus (hRSV), human parechoviruses (hPeVs), adenoviruses (AdVs) and influenza A and B viruses (FLU) as described previously.^14^, ^16^ HRV typing was done using a nested RT‐PCR assay targeting the VP4/VP2 domains.^17^

The HRV evolutionary history was inferred using a Neighbor‐Joining algorithm, a p‐distance substitution model and a complete deletion option in MEGA6 (www.megasoftware.net/) on a data set comprising 434 nucleotides from the viral VP2/VP4 domains after deletion of 5′‐untranslated region sequence portions. Recombination in the data set was discarded using RDP V4.95. Only HRV was typed due to frequent detection and rapid evolution allowing sufficient phylogenomic resolution.

Statistical analysis was performed by using SPSS 13.0 (IBM) with *Χ* ^2^ or Fisher's test when analyzing numbers less than 5 in any cell. Confidence intervals were calculated using Open Epi (www.openepi.com) using the Wilson method.

## 3 RESULTS

Many epidemiological features of respiratory viruses in our study were similar to those from other reports worldwide. The first similarity was a high overall virus detection rate of 65.2% (184 of 282 patients) together with the genetic diversity of the respiratory pathogens, including all viruses that were tested except hMPV (Table 1). Both attributes were previously reported in studies conducted in temperate and tropical regions of Brazil (~60.0%),^18^, ^19^ in a slum community from Kenya (71.0%),^20^ and in a German cohort (56.6%).^14^

**Table 1:** Virus detection rates

Virus	N‐positive patients	Detection rate^a^ %	95% Confidence interval
HRV	93	33.0	27.7‐38.7
HRV A	41	14.5	10.9‐19.1
HRV B	5	1.8	0.8‐4.1
HRV C	31	11.0	7.9‐15.2
HRV untyped	16	5.7	3.5‐9.0
hRSV	34	12.1	8.7‐16.4
hPiV^b^	27	9.6	6.7‐13.6
hPiV‐1	1	0.3	0.1‐2.0
hPiV‐2	3	1.1	0.4‐3.1
hPiV‐3	16	5.7	3.5‐9.0
hPiV‐4	8	2.8	1.4‐5.5
HCoV^b^	26	9.2	6.4‐13.2
HCoV‐229E	8	2.8	1.4‐5.5
HCoV‐NL63	4	1.4	0.6‐3.6
HCoV‐OC43	5	1.8	0.8‐4.1
HCoV‐HKU‐1	17	6.0	3.8‐9.4
EV	26	9.2	6.4‐13.2
AdV	18	6.4	4.1‐9.9
FLU	13	4.6	2.7‐7.7
FLU‐A	8	2.8	1.5‐5.5
FLU‐B	5	1.8	0.8‐4.1
hPeV	6	2.1	1.0‐4.6
Total number of positive patients^b^/number of patients tested	184/282	65.2	59.5‐70.6

Abbreviations: AdV, adenovirus; EV, enterovirus; FLU, influenza A and B viruses; HCoV, human coronaviruses 229E, NL63, OC43 and HKU1; hPeV, human parechovirus; hPIV, human parainfluenzavirus; hRSV, human respiratory syncytial virus; HRV, human rhinoviruses A‐C.

^a^ Detection rates were calculated as the fraction of patients infected and the total study population.

^b^ Because of coinfections in several patients, the number of individual virus detections (N = 252), including HCoV (N = 34) and hPiV (N = 28), were higher than the number of infected patients.

The second similarity was the predominance of HRV (33.0%) and hRSV (12.1%) detections (Table 1), also observed in hospitalized patients in distinct cities of Brazil, in a slum community in Kenya^18^, ^19^, ^20^ and in German children.^14^

The third similarity was the high rate of viral coinfections (28.3%) (Figure 1B) which was similar to rates observed in a study from Curitiba, southern Brazil (29.0%)^19^ and in a Kenyan slum community (27.0%).^20^ Our data also point to the predominance of HRV in cases of coinfections (30 of 52 coinfections, 58.0%), a feature similarly reported in a study conducted in Brazil (69.0% of all coinfections)^19^ that is likely influenced by the overall high number of HRV infections. Albeit not statistically significant, HRV, hRSV, and FLU were commonly detected as monoinfections (67.7%, 73.5%, and 76.9% respectively; *P* > .05 for all three viruses), whereas HCoV, AdV, hPiV, EV, and hPeV were significantly more frequently detected as coinfections (53.8%, 61.1%, 66.7%, 80.8%, and 83.3%, respectively; *P* < .05 for all) (Figure 1C). These data were consistent with results of a study including data from eight tropical countries showing that HRV, hRSV, and FLU were more commonly detected as monoinfections, AdV as coinfections and HCoV and hPiV equally distributed between both coinfections and monoinfections.^21^ Similarly, preliminary studies from Brazil showed that hRSV and FLU were most frequently detected as monoinfections and EV and AdV as coinfections.^13^, ^19^

The fourth similarity was the seasonal variation of hRSV and HCoV detections. Particularly, hRSVs were more frequently detected during autumn (Fisher's exact, *P* = .004), a pattern already observed in surveillance studies carried out in two distinct São Paulo city hospitals.^22^, ^23^ HCoV were more frequently detected during winter (Fisher's exact, *P* = .011; Figure 1D), as observed previously in a study conducted during 20 years in the United States.^24^

The fifth similarity included statistically significant associations of FLU and hRSV infections with more severe symptoms such as fever (Fisher's exact, *P* = .007 and *P* = .02, respectively) and of hRSV infections with dyspnea (Fisher's exact, *P* = .038) (Figure 1E). The association of hRSV infections with more severe symptoms was consistent with a previous study from Brazil showing higher probability of children infected by hRSV to present more severe disease compared to infections with other respiratory viruses.^13^ Together with the low frequency of codetections of FLU and hRSV, this feature is consistent with higher pathogenicity of both viruses.^18^ In contrast to hRSV, HRV infection was significantly less frequently associated with fever and dyspnea (Fisher's exact, *P* = .001, *Χ* ^2^ *P* = .005) (Figure 1E). The lower proportion of HRV infections and cases of fever compared to other community‐acquired respiratory virus infections was similar to a previous study conducted in patients hospitalized with ARI in Curitiba, southern Brazil.^19^

The sixth similarity was a predominance of hRSV and hPeV detections in patients aged ≤1 year (Fisher's exact, *P* = .001 and *P* = .01, respectively) (Figure 1F). Similarly, Annan et al^14^ reported that pneumoviruses including hRSV were more frequently detected at younger ages in cohorts from Ghana and Germany, predominantly in patients less than 1 year of age.

The seventh similarity comprised two contrasting HRV epidemiological patterns, namely replacement of some HRV strains and maintenance of other HRV strains over time. HRV comprises three defined species termed HRV A‐C, and >100 different types likely representing multiple distinct serotypes.^25^ We successfully typed a total of 77 HRV strains representing all three HRV species (GenBank accession numbers MH824434‐MH824510), whereas 16 HRV strains could not be typed. Proportions of individual HRV species in our study (53.2% HRV‐A, 6.5% HRV‐B, 40.3% HRV‐C) were in agreement with other reports worldwide.^17^, ^26^ The majority of distinct HRV strains in our study were detected only in one season, a common HRV epidemiological pattern.^17^, ^25^, ^26^, ^27^ In contrast, seven clusters termed I‐VII, each composed of three to five patient‐derived HRV strains presenting a very low mutual pairwise sequence distance (≤2%), were detected over more than two seasons. The seven clusters belonged to HRV species A and C and differed from one another by 15% to 39% mutual nucleotide sequence distance. Previous studies on HRV typing using the genomic fragment used in our study found that defined HRV serotypes differed by at least 10% mutual nucleotide sequence distance.^17^ Following this criterion, strains belonging to one cluster in our study thus likely represent the same HRV serotype, whereas the seven clusters all represent distinct HRV serotypes. Community protective immunity can dramatically limit the circulation of defined viral serotypes, including HRV and other viruses.^17^, ^28^ This was apparently the case for some, but not all HRV serotypes in our study, the latter potentially facilitated by the population structure of the slum. Prolonged circulation of HRV clusters was detected specifically during winter and spring (Figure 1G, clusters I, III, V, VII), summer and spring (cluster II), winter and summer (cluster IV), and autumn and summer (cluster VI). These data were reminiscent of prolonged circulation of closely related HRV lineages for 4 to 12 months or over three consecutive seasons reported previously from Sweden and Finland^26^, ^27^ and are thus not unique to our study setting. Notably, we cannot exclude that HRV strains may have been re‐introduced repeatedly from other areas of São Paulo, a large metropolis accumulating about 21 million inhabitants, potentially facilitated by commuting of slum inhabitants to nearby areas for labor.^15^

We observed only two potentially unusual patterns including the total absence of hMPV detections and absence of FLU seasonality. The first may be explained by widely documented local variation in annual hMPV circulation,^29^ exacerbated here by limited sampling during the expected hMPV season, usually late winter/early spring as reported previously in São Paulo.^30^ The absence of FLU seasonality, usually related to rainy seasons in tropical and subtropical regions,^31^ may have been facilitated by the population characteristics of the Brazilian slum allowing continued FLU transmission and maintenance over relatively dryer seasons. However, a similar lack of seasonality was not observed for hRSV and HCoV, suggesting that definite assertions on the reasons underlying the absence of FLU seasonality cannot be made at this point.

## 4 DISCUSSION

Our study confirmed the importance of respiratory viruses in children with ARI inhabiting a slum in São Paulo, Brazil, a densely populated area with subtropical climate.

Despite the extremely high population density of the slum, most virus‐associated epidemiological patterns were surprisingly similar to others reports, including overall virus detection rates, the predominance of HRV and hRSV and seasonal variation of different viruses.^18^, ^19^, ^21^, ^22^, ^23^, ^25^ More specifically, usage of identical methodology enables direct comparisons between this study and our previous investigation of a rural area in Ghana and an urban environment in Germany.^14^ Again, frequent detection of hRSV and HRV, viral genetic diversity, seasonal patterns, and levels of coinfections were roughly comparable between Brazil, Ghana, and Germany. This is particularly noteworthy because the population density of the Brazilian slum is close to 600‐fold higher than in the African site and 25‐fold higher than in the European site.

Our study has several limitations, including (a) a relatively small sample size, (b) lack of a control group of children with ARI sampled simultaneously in non‐slum areas, (c) sampling over only 1 year, since respiratory virus epidemiological patterns may greatly vary over time, (d) absence of information regarding daycare attendance and influenza vaccination status, (e)​​​​ sampling of only one slum community, preventing generalization of results and (f)​​​​ the absence of screening for bacterial pathogens, such as *S. pneumoniae*. The strengths of our study include (a) sampling in an area of difficult access, (b) sensitive methodology targeting all major respiratory viruses, (c) contiguous sample collection over 1 year, and (d) the combination of clinical and virological data.

Our data indicate that the epidemiology of virus‐associated ARI is not consistently affected by SES, population density, and climatic factors. Multi‐centric epidemiological studies conducted over several consecutive years from multiple areas representing replicates of climatic, SES‐ and population‐associated conditions, analyzed with identical quality‐controlled methodology would be desirable to better understand respiratory virus infection patterns and potential effect modifiers. Meta‐analyses of the vast literature describing viral ARI epidemiology worldwide may provide cost‐efficient alternative approaches, yet studies focusing on respiratory virus epidemiology in slum populations are scarce compared to studies from affluent settings.^32^, ^33^ This knowledge will be crucial to inform potential public health interventions to reduce disease burden in the population.
